# Vibrational Spectra Study of the Interactions Between Keggin Heteropolyanions and Amino Acids

**DOI:** 10.3390/molecules14093214

**Published:** 2009-08-27

**Authors:** Fatemeh F. Bamoharram

**Affiliations:** Department of Chemistry, Islamic Azad University, Mashhad -Branch, Mashhad, Iran; E-mails: abamoharram@yahoo.com or fbamoharram@mshdiau.ac.ir

**Keywords:** heteropolyanion, amino acid, vibrational spectra, Keggin

## Abstract

A study of the effects of the interactions with amino acids on the vibrational spectra of Keggin heteropolyanions (Na_n_H_3-n_[XM_12_O_40_]^n-^; n = 0,1,2,3; X = P^V^, Si^IV^, M = W^VI^, Mo^VI^) has been performed. Interactions occurred for all of the studied compounds leading to changes and splits in the stretching frequencies. Depending on the amino acid type, the IR bands for interbridge stretchings (M-O_b_-M) and terminal stretchings (M-O_d_) were affected.

## 1. Introduction

Heteropolyanions (HPAs) are widely used as solid and green catalysts for fine organic synthetic processes [1,2,3,4,5,6,7,8], corrosion resistant coatings, dopants in sol – gel matrices, membranes in selective electrodes, in gas detection apparatus, in solid-state electrochromic devices, etc [9,10,11,12]. However, although many HPAs have been shown to be biologically active, their biological properties have not been investigated much. To date two types of activity − antiviral and, to a lesser extent, antitumoral − have dominated their medicinal chemistry [13]. In the first area the effectiveness of these compounds against several viruses such as vesicular stomatitis, polio, rubella, rauscher leukemia, rabies, etc, has been studied [13].

The reasons for the biological activity of HPAs may include ionic size and charge, electron-transfer properties, the behavior of polyanions in extremely dilute solutions at physiological pH, etc. [14]. Because HPAs are potent inhibitors of reverse transcriptase and other related enzymes [15], understanding of the nature of the interaction of these compounds with other biologically relevant compounds such as proteins has been an active focus of research. The anti-viral activity of this kind of polyanions is the result of their interactions with viral enzymes or, as recently determined, related with their ability to bind to the viral cell envelope, and perhaps not with their ability to inhibit viral reverse transcriptase, as thought before [16,17].

In order to shed more light on this action, we present here some results obtained a study of the interactions of various heteropolyanions, related to the Keggin structure, with different amino acids such as glycine, phenylalanine, valine, and leucine. HPAs are excellent molecular acceptors for a number of molecular donor organic substrates containing N, S, O atoms, i.e. amino acids [18,19], octohydroquinoline [20], and crown ethers [21].

A literature survey showed that, although the interaction of HPAs with amino acids has been studied with different methods, the role of (*S*)-(-)-amino acids, has been largely overlooked, so the novelty of this work lies in selection of (*S*)-(-)- amino acids as a study topic, and to the best of our knowledge, no systematic study via FTIR spectroscopy of the interactions between Keggin HPAs (as pure acid and salt) with these amino acids has been reported so far. Usually FTIR spectroscopy can be used to confirm the interaction of HPAs with different molecules [22]. My goal was to show and prove the initial interaction of HPAs with amino acids as the basic units of biological molecules in order to assist in development of future compounds with selective affinity for particular amino acids. In other words, I was looking for the answer to this question: Is there initial interaction between the selected HPAs and amino acids? Using amino acids as counterions, I have undertaken a systematic vibrational study of the interactions of HPAs with amino acids and showed that intermolecular interactions led to a change in the frequencies of the metal–oxygen stretching bands. The results of this study should be useful to other researchers for further investigations and could be extended from nitrogen-containing molecules to sulfur- and oxygen-containing molecules.

## 2. Results and Discussion

The influence of counterions on the vibrational spectra of complex anions in the solid state has been widely studied using several kinds of approaches. One of them, based on the factor group approximation, considers the coupling between formula units in the Bravis cell (without regard to the nature of the coupling), which supposes some previous knowledge of the crystal structure of the studied compound. Another more chemical approach considers the anion-cation interactions as perturbations on the charge-density repartition, on the geometry of the anion, and consequently, on the vibrational spectra with respect to the isolated anion. This approach does not need any prior knowledge of the crystal structure and can provide information about intermolecular interactions [23].

In this study, the interaction of amino acids as counterions such as glycine, phenylalanine, valine, and leucine, with Keggin heteroppoly anions, Na_n_H_3-n_[XM_12_O_40_]^n-^ (n = 0,1,2,3, X = P^v^, Si^IV^, M = W^VI^, Mo^VI^), were studied using vibrational spectroscopy. The IR frequencies were studied after preparation and isolation of possible adducts in our laboratory according to the procedures described in the Experimental section. The frequencies discussed are listed in Table 1, along with the corresponding assignments.

**Table 1:** Assignments in the vibration spectra of Keggin HPAs with amino acids as counterions.

Entry	HPA and HPA with amino acid	Vibrational frequencies
1	H_3_[PW_12_O_40_]	1080(s), 990(sh), 982(s), 890(s), 810(vs), 597(w), 527(m)
2	Na_3_[PW_12_O_40_]	1081(s), 995(sh), 982(s), 922(m), 900(m) , 805(vs), 592(w), 522(sh)
3	H_4_[SiW_12_O_40_]	1020(w), 981(s), 928(vs), 880(m), 785(vs), 552(sh), 540(m)
4	Na_2_H[PMo_12_O_40_]	1068(s), 978(sh), 962(vs), 869(s), 785(vs), 593(w)
5	H_3_[PMo_12_O_40_]	1064(s), 975(sh), 963(vs), 870(s), 810(sh), 785(vs), 593(w)
6	H_4_[SiMo_12_O_40_]	995(sh), 957(s), 904(vs), 855(m),770(vs), 535(m), 442(w)
7	[leucine]_x_H_y _[PMo_12_O_40_]	1061.8(s), 973.4(sh), 961.4(vs), 867(w), 891(s), 810(sh), 794(vs), 739.1(sh), 677.3(sh), 668.7(s), 633.5(sh)
8	[phenylalanine]_x_H_y_[PMo_12_O_40_]	1062.7(s), 975(sh), 966.5(vs), 892(sh), 871.3(w), 889(s), 810(sh), 795(vs), 739.1(sh), 605.7(sh), 669.6(m)
9	[glycine]_x_H_y_[PMo_12_O_40_]	977.7(w), 975(sh), 955.4(w), 909(vw), 925.3(w), 835.2(sh), 732(w), 668.7(s)
10	[phenylalanine]_x_ Na_y_[PMo_12_O_40_]	1061.8(vs), 975(sh), 967(vs), 891(s), 871.4(w), 800.6(vs), 741.7(vw), 668.2(s), 608.5(sh)
11	[valine]_x_H_y_ [PMo_12_O_40_]	1062.7(s), 973.4(sh), 963.1(vs), 886.7(m), 867(m), 810(sh), 795.9(sh), 790.3(vs), 740.8(sh), 667.8(s)
12	[leucine]_x_Na_y_[PMo_12_O_40_]	1064.4(vs), 970(vs), 959.7(s), 896.1(s), 857.5(w), 791.9(vs), 740(sh), 673.8(sh), 668.7(s), 636.1(w)
13	[glycine]_x_Na_y_[PMo_12_O_40_]	1116.8(vw), 976.9(w), 976.9(w), 954.5(s), 922.7(s), 908.2(w), 836.9(sh), 733.1(sh), 675.6(sh), 667.8(s), 610.3(sh)
14	[valine]_x_H_y_[PW_12_O_40_]	1080.7(vs), 990(sh), 982.8(vs), 995(sh), 893.6(vs), 811.2(vs), 738.2(w) 668.7(s)
15	[phenylalanine]_x_H_y_[PW_12_O_40_]	1061.3(w), 976.9(s), 990(sh), 924.5(s) 880(sh), 794(s)
16	[leucine]_x_H_y_[SiW_12_O_40_]	1133.1(w), 975.1(vs), 1015.5(s), 975.1(vs), 985.1(sh), 922.7(vs), 890.1(w), 794(b), 667.8(s), 635.2(w)
17	[valine]_x_H_y _[SiW_12_O_40_]	1016.3(s), 971.7(vs), 981(sh), 924.5(vs). 888.4(w), 867(w), 794(vs), 679(sh), 667.8(s), 635.2(w)
18	[glycine]_x_H_y_[SiW_12_O_40_]	1014.6(s), 972.5(vs), 989(sh), 925(vs), 935.3(sh), 889.3(w), 788.9(vs), 667.8(s)
19	[glycine]_x_H_y_[SiMo_12_O_40_]	1073.7(vw), 976.4(m), 896.2(vw), 837.3(w), 732.2(w), 678.5(sh), 669.1(s)
20	[valine]_x_H_y_[SiMo_12_O_40_]	1079(vw), 976.9(w), 897.9(w), 834.4(w), 733.1(w),668.7(s), 615.5(sh)
21	[leucine]_x_H_y_[SiMo_12_O_40_]	1063.5(vw), 976.9(w), 904.7(w), 790.6(vw), 737.4(w), 668.7(s), 614.6(sh)
22	[phenylalanine]_x_H_y_[SiW_12_O_40_]	1071.2(sh), 960.9(s), 920.9(s), 891(sh), 880.9(w), 747.8(w), 668(s)
23	[glycine] _x_H_y_[PW_12_O_40_]	976.9(w), 900(w), 838.6(vw), 830.1(vw), 788(w), 733.9(m), 668.7(s)
24	[leucine]_x_H_y_[PW_12_O_40_]	1081.3(vs), 979.4(s), 896.5(s), 807(w), 740.8(w), 678.8(sh), 668.6(s)

It is suggested that in the studied amino acids the relative position of COOH and NH_2_ groups is very important, as these functional groups should be adjacent for interaction to exist. From the information in Table 1, it can be seen the intermolecular interactions between the oxygens of the polyanions and amino acid nitrogens led to changes in the intensity and position of the corresponding IR bands. 

The [XM_12_O_40_](Keggin structure) consists of one XO_4_ tetrahedron (Y = Si^IV^, Ge^IV^, P^V^, …) surrounded by four M_3_O_13_ sets (M = W^VI^, Mo^VI^) linked together through oxygen atoms (named M-O_b_-M). The YO_4_ tetrahedron is assumed to vibrate almost independently from the rest of the anion. This assumption is particularly valuable for X = P^V^. The symmetric and asymmetric stretching of the different kinds of M-O bonds are observed in the following spectral regions: M-O_d_ bonds (1,000-960 cm^-1^), M-O_b_-M bridges (inter bridges between corner-sharing octahedra) (890-850 cm^-1^), M-O_c_-M bridges ("intra" bridges between edge-sharing octahedra, (800-760 cm^-1^). Only the M-O_d_ stretching can be considered as pure vibrations: the stretching involving O_b_ or O_c_ atoms present some bend character [24,25].

**Figure 1:**
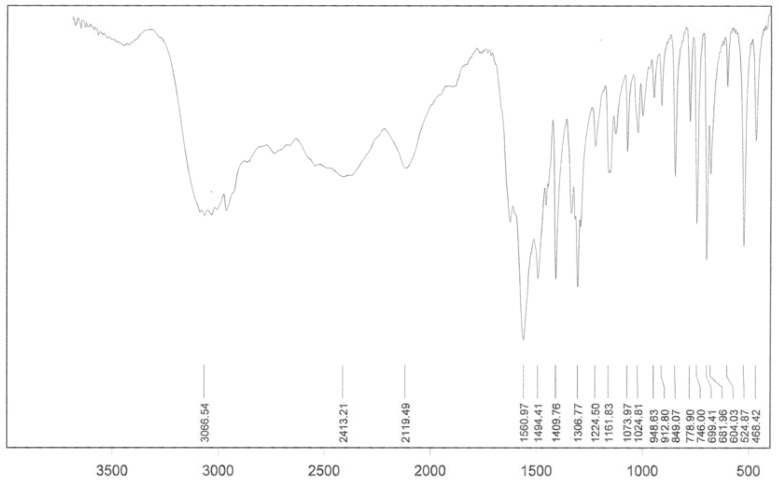
IR spectrum of phenyalanine.

**Figure 2:**
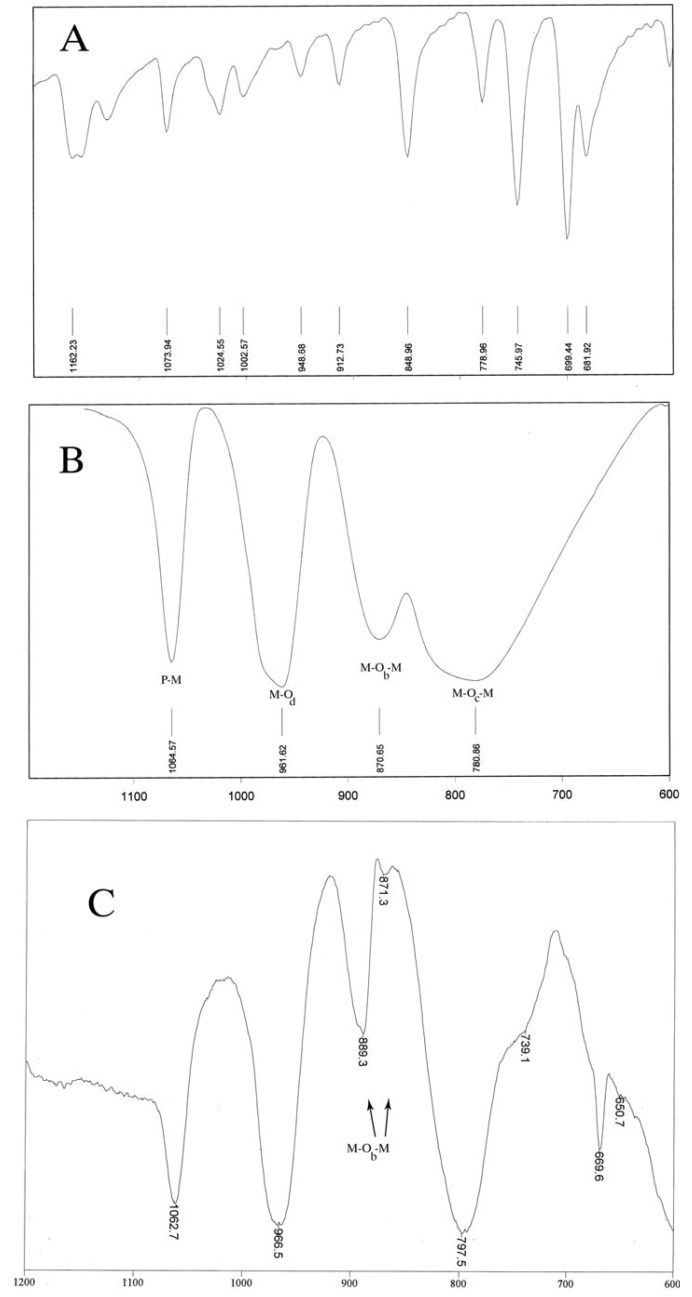
IR spectra of phenyalanine (A), H_3_PMo_12_O_40 _(B), phenylalanine and H_3_PMo_12_O_40 _ (C).

In our study, the IR spectrum of the present compounds exhibits the characteristic frequencies of the Keggin structure in the range 1,100-600 cm^-1^. Compared with the initial Keggin structures (entries 1-6), the bands arising from the HPAs change obviously either in intensity, or in position. Comparing the IR spectrum of the prepared compounds (entries 7-13) with the IR spectrum of H_3_[PMo_12_O_40_], the vibrational band of the M-O_b_-M of the present compound is split from 870 into two bands, due to difference in the M-O_b_-M bonds. As a typical spectrum, the IR of the interaction of H_3_[PMo_12_O_40_] and phenylalanine is shown in Figure 2. Figure 1 shows the IR spectrum of phenylalanine alone. In Figure 2 for a correct comparison, the range of 600-1,200 cm^-1^ has been selected for phenylalanine (A), H_3_[PMo_12_O_40_] (B), and H_3_[PMo_12_O_40_] with phenylalanine (C). This range is related to the fingerprint region of POMs.

For H_4_[SiMo_12_O_40_] and H_3_[PW_12_O_40_] the vibrational band of the W-O_d_ is split into two bands, from 981 and 982-990, respectively, due to the differences in the 12 W-O_d_ bonds. This means that, in H_3_[PMo_12_O_40_] the inter bridges between corner-sharing octahedra (890-850 cm^-1^) have been affected, while in H_3_[PW_12_O_40_] and H_4_[SiW_12_O_40_] the M-O_d_ stretchings have been affected. It is suggested that i) the central transition metal can be affected the position of interaction, and ii) interaction of amino acids has occurred with selective affinity.

In addition our findings show that many of the vibrational bands of HPAs have blue-shifted and many of them have red-shifted, indicating that many of the bonds were strengthened and the others were weakened. This means that the HPA has a distorting effect owing to the interaction between the nitrogen atom of the amino acid and the oxygen atom of the HPA. Probably, the extent of distortion is roughly parallel to the volumes of the organic molecules [26]. The strong band at 3,454 cm^-1^ (this band was observed in all of the IR spectra) is assigned to the water molecules involved in hydrogen bond interactions with HPA and the amino acid molecules to form an extensive three dimensional structure [27]. The hydrogen bond can be formed via the protonated amino acid.

The obtained IR spectra for the interactions between H_4 _[SiW_12_O_40_] with phenylalanine (Entry 22), H_4_[SiMo_12_O_40_] with luceine, valine and glycine (Entries 19-21) and H_3_[PW_12_O_40 _] with glycine and luceine (Entries 23, 24) show a strong deformation. This indicates that the HPA has a large distorting effect owning to the interaction between the amino acid and HPA.

In conclusion, the results described in this paper strongly indicate that the HPAs have a strong interaction with amino acids. Our results show that bond type in HPAs, as well as position of functional groups in amino acids are important factors. Obtained results in this study are significant for biochemistry, clinical chemistry and analytical chemistry and can be developed for different viruses with different protein envelopes.

## 3. Experimental

### 3.1. General

All of the heteropoly anions and the amino acids were purchased from commercial sources. FTIR spectra were recorded with a Bruker 500 scientific spectrometer as KBr pellets.

### 3.2. General procedure for the preparation of amino acid-heteropolyanion adducts

In a typical reaction, amino acid (0.5 g) was dissolved in a 1M HCl solution (10 mL) and solid heteropolyanion (3 g) was then added and dissolved with stirring for 3 hours. The solution was placed in a vacuum dessicator. After 5-7 days later the produced products have been formed as solid products. These products were filtered and dried in air. Then were analyzed by IR spectroscopy. These results are shown in Table 1.
